# Evisceration and enucleation cases in the ophthalmologic emergency department of a tertiary Brazilian hospital

**DOI:** 10.5935/0004-2749.20220073

**Published:** 2025-02-11

**Authors:** Camila Kase, Luis Filipe Nakayama, Vinicius Campos Bergamo, Nilva Simeren Bueno de Moraes

**Affiliations:** 1 Departament of Ophthalmology, Escola Paulista de Medicina, Universidade Federal de São Paulo, São Paulo, SP, Brazil

**Keywords:** Eye evisceration, Eye enucleation, Corneal ulcer/ epidemiology, Endophthalmitis, Eye injuries, Emergency medical services, Eye health services, Evisceração do olho, Enucleação ocular, Úlcera da córnea/epidemiologia, Endoftalmite, Traumatismos oculares, Serviços médicos de emergência, Serviços de saúde ocular.

## Abstract

**Purpose:**

To analyze the epidemiological profiles of evisceration and enucleation cases in the ophthalmologic emergency department of a Brazilian tertiary hospital.

**Methods:**

Patients treated in the ophthalmologic emergency department of Hospital São Paulo (Universidade Federal de São Paulo) during the period 2013 to 2018 were retrospectively evaluated. Urgent cases of evisceration or enucleation surgery were included, and elective cases were excluded. The following information was extracted from the patients’ medical records: demographic data, immediate and associated reasons for the surgical procedure, informed visual acuity, symptom duration until ophthalmologic care, complications, distance from the residence to the tertiary hospital, and time of hospitalization.

**Results:**

In total, 61 enucleations and 121 eviscerations were included in this study. The patients had a mean age of 63.27 ± 18.68 years. Of the patients, 99 were male (54.40%), and 83 were female (45.60%). The indications for evisceration or enucleation were corneal perforation with (44.50%) and without (23.63%) signs of infection, endophthalmitis (15.38%), ocular trauma (14.29%), neoplasia (0.55%), burn accident (1.10%), and *phthisis bulbi* (0.55%). The self-reported visual acuity was no light perception (87.36%) or light perception (1.10%). However, 3.30% of the patients did not cooperate with the examination, and no information on visual acuity was available for the remaining 8.24%. The mean symptom duration before ophthalmologic care was sought was 18.32 days. Two patients had sympathetic ophthalmia after evisceration.

**Conclusions:**

More eviscerations than enucleations were performed throughout the study period. The most common demographic characteristics were age >60 years and male sex. The main indications for urgent evisceration and enucleation procedures were corneal perforation with and without infection, endophthalmitis, and ocular trauma. The findings from this study could guide clinicians in performing preventive measures to avoid destructive eye procedures.

## Abstract (pt)

**Objetivo:**

Analisar o perfil epidemiológico dos casos de evisceração e enucleação no pronto-socorro oftalmológico de um hospital terciário brasileiro.

**Métodos:**

Análise retrospectiva dos casos tratados no pronto-socorro oftalmológico do Hospital São Paulo (Universidade Federal de São Paulo) entre os anos de 2013 a 2018. Os casos urgentes de evisceração e enucleação foram incluídos e os casos eletivos foram excluídos. A análise dos prontuários médicos foi baseada em: dados demográficos, causas imediatas e associadas ao procedimento, acuidade visual informada, duração dos sintomas antes do atendimento oftalmológico, complicações, distância da residência até o hospital e tempo de hospitalização.

**Resultados:**

61 enucleações e 121 eviscerações foram incluídas no estudo. Os pacientes tinham uma média de idade de 63,27 ± 18,68 anos; 99 eram do sexo masculino (54,50%) e 83 do sexo feminino (45,60%). As indicações de evisceração e enucleação foram: perfuração corneana com (44,50%) e sem (23,63%) sinais infecciosos, endoftalmite (15,38%), trauma ocular (14,29%), neoplasia (0,55%), queimadura (1,10%) e *phthisis bulbi* (0,55%). A acuidade visual informada foi de ausência de percepção luminosa (87,36%), percepção luminosa (1.10%), ausência de colaboração (3,30%) e sem dados informados (8,24%). A média de tempo até a busca pelo serviço oftalmológico foi de 18,32 dias. Houve 2 casos de oftalmia simpática após evisceração.

**Conclusões:**

Eviscerações foram predominantemente realizadas em comparação a enucleações em todo o período de estudo. As características demográficas mais comuns foram idade >60 anos e sexo masculino. As principais indicações para procedimentos urgentes de evisceração e enucleação foram perfuração corneana com e sem infecção, endoftalmite e trauma ocular. Este estudo poderia guiar medidas preventivas para evitar procedimentos oculares destrutivos.

## INTRODUCTION

Evisceration and enucleation are procedures that lead to severe changes in the orbital structure and function. Evisceration removes the content of the eyeball while preserving the conjunctiva, tenon capsule, sclera, extraocular muscles, optic nerve, and in some cases, the cornea. By contrast, enucleation removes the eyeball completely after isolation and sectioning of the extraocular muscles and optic nerve^(1)^.

The main indications for destructive ocular surgeries include conditions of poor visual prognosis associated with severe ocular trauma, ocular infection, painful blind eye, advanced glaucoma, intraocular neoplasia, and *phthisis bulbi*^(1-3)^. The absolute contraindication to evisceration surgery is suspected or confirmed intraocular tumor^(2)^.

Some authors have reported an increase in the number of indications for evisceration instead of enucleation^(3-5)^. The benefits of evisceration are better cosmesis and functionality and shorter operative time, with less exposure to anesthesia. In addition, evisceration is similar to enucleation in terms of pain relief, infection treatment, and improved appearance^(2,3,6)^. The main concern in performing evisceration surgery is the risk of sympathetic ophthalmia, but previous studies showed that this complication is infrequent^(2,7,8)^. As both procedures are debilitating, family involvement and psychological support throughout the preoperative and postoperative process and an explanation of the possibility of prosthetic adaptation are important^(1)^.

In the present study, our objective was to analyze epidemiological data on emergency cases managed with ocular enucleation or evisceration.

## METHODS

This study was a retrospective observational and descriptive analysis of the medical records of patients evaluated in the ophthalmologic emergency department of Hospital São Paulo, Federal University of São Paulo (Brazil). The study was approved by the institutional ethics committee of Universidade Federal de São Paulo (UNIFESP approval number: 1271/2018) and conducted in accordance with the principles of the Declaration of Helsinki.

The period of analysis was January 2013 to September 2018. Patients who visited the ophthalmologic emergency department and were admitted for urgent surgical procedures with indications for enucleation or evisceration were included. Patients hospitalized for elective surgical procedures were excluded. Orbital computed tomography was performed when opaque media did not permit observation of the eye fundus to rule out intraocular neoplasia before surgery and to assess signs of orbital fracture in patients with severe eye trauma and potential orbital extension in patients with eye infection. If available, ocular ultrasonography was performed to identify intraocular changes before surgery and if intraocular neoplasia was suspected.

All the patients were informed about the possible surgical and postoperative complications and signed an informed consent form. All the surgical procedures were performed under general anesthesia. All the patients were discharged from the hospital with a prescription of an antibiotic combined with corticosteroid ointment or eye drops. Orbital cellulitis was managed with specific antibiotic treatment. Suspected intraocular neoplasia was assessed by an ocular oncologist before the procedure, and follow-up was conducted by the ocular oncology team.

### Enucleation

Enucleation surgery was performed with a 360° conjunctival peritomy and conjunctiva and tenon capsule dissections. The extraocular muscles were isolated and sectioned at the point of insertion, followed by sectioning of the optic nerve with blunt scissors. After hemostasis, the conjunctiva and tenon capsule were sutured with Polyglactin 910 6-0.

### Evisceration

Evisceration surgery was performed with a 360° conjunctival peritomy, dissections of the conjunctiva and tenon, and perilimbal corneal incision for removal of the cornea. Curettage of all intraocular materials was performed, followed by temporary application of absolute intraocular alcohol, hemostasis, scleral suture with Nylon 6-0, and tenon and conjunctival suture with Polyglactin 910 6-0.

The following epidemiological data were extracted from the patients’ medical records: age, sex, eviscerated or enucleated eye, immediate cause, associated causes, self-reported visual acuity, time between symptom onset and seeking of eye care, complications, distance from residence to tertiary hospital, and time of hospitalization.

Data were input into Excel v16. 16.5 (Microsoft, Redmond, WA) and exported to IBM SPSS Statistics for Windows (IBM Corp., released 2017, Version 25.0, Armonk, NY) for statistical analysis. Descriptive analysis was performed, and means, standard deviations, and 95% confidence intervals were calculated.

## RESULTS

During the period of January 2013 to September 2018, 182 patients were admitted for urgent evisceration or enucleation at Hospital São Paulo. During this period, 326,866 consultations were performed in the ophthalmologic emergency department, with an incidence of evisceration or enucleation of 0.06%.

In total, 61 enucleations were performed in 61 patients (32 males [52.46%] and 29 females [47.54%]), and 121 eviscerations were performed in 121 patients (67 males [55.37%] and 54 females [44.63%]). The mean age of the patients included in the study was 63.27 years, with a range of 14 to 95 years and a standard deviation of 18.68. Surgery was performed in the left eye in 102 patients (56.04%) and the right eye in 80 patients (43.96%).

**Figure 1:**
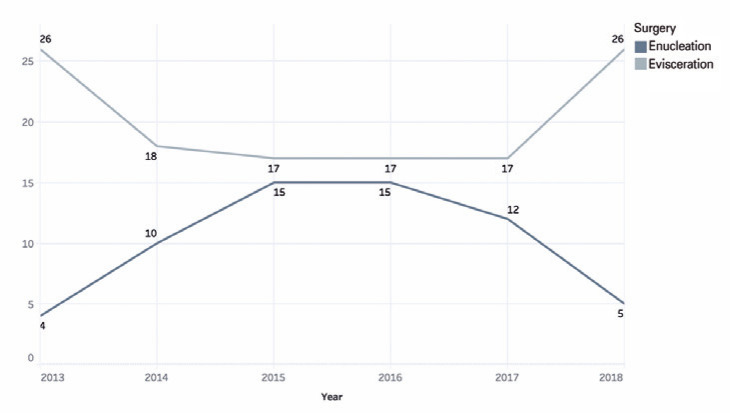
Number of eviscerations and enucleations during the period from 2013 to 2018.

In 2013, more eviscerations than enucleations were performed. In 2014 and 2015, the number of enucleations increased in association with a decrease in the number of eviscerations, followed by a decrease in enucleations and increase in eviscerations in subsequent years (Figure 1).

**Figure 2:**
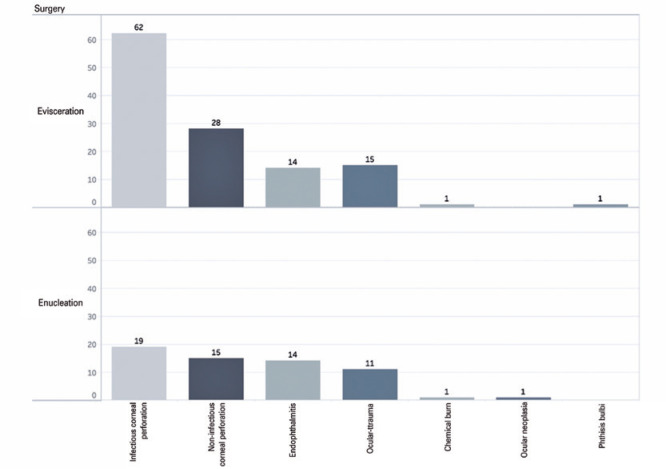
Distribution of the main indications of evisceration and enucleation during the period from 2013 to 2018.

The indications for evisceration or enucleation were as follows: corneal perforation with (n=81, 44.50%) and without (n=43, 23.63%) signs of infection, endophthalmitis (n=28, 15.38%), ocular trauma (n=26, 14.29%), neoplasia (n=1, 0.55%), burn accident (n=2, 1.10%), and *phthisis bulbi* (n=1, 0.55%). The main indications for evisceration were corneal perforation with (n=62) or without (n=28) infection, ocular trauma (n=15), endophthalmitis (n=14), chemical burn (n=1), and *phthisis bulbi* (n=1). For enucleation, the main indications were corneal perforation with (n=19) and without (n=15) infection, endophthalmitis (n=14), ocular trauma (n=11), ocular neoplasia (n=1), and chemical burn (n=1; Figure 2).

The intraocular content and ocular globe were sent to the pathology department for analysis in 66.12% of the evisceration cases and 81.97% of the enucleation cases, respectively.

Orbital computed tomography was performed for 129 patients (70.88%); and ocular ultrasonography, for 22 patients (12.09%). Thirty-one patients (17.03%) did not undergo orbital or ocular imaging because neoplasia was ruled out by ophthalmologic examination, no risk of orbital extension of ocular infection was found (6.20%), or assessment of orbital fracture was unnecessary because the eye trauma did not affect adjacent tissues (1.65%). The medical records of 14 patients (7.69%) did not provide an explanation of why imaging examinations were not requested. The patients who underwent orbital computed tomography showed no signs of intraocular neoplasia; 9.30% had signs of orbital cellulitis requiring intravenous antibiotic therapy, and 5.43% had signs of orbital fracture after traumatic injury. Suspicion of choroidal melanoma was reported in one patient after ocular ultrasonography.

The mean time elapsed between the onset of symptoms and demand for eye care was 18.32 days (1-720 days). The mean distance between the residence and care unit was 28.04 km (1-653 km), and the mean hospitalization time was 3 days (1-35 days).

### Visual acuity

Most patients presented a visual acuity of no light perception (NLP) in the initial evaluation (87.36%). In two patients (1.10%), the initial visual acuity was light perception (LP), and none had visual acuity better than hand motion. Six patients (3.30%) did not cooperate with the examination or respond owing to cognitive disorders. The medical records of 15 patients (8.24%) did not include information on visual acuity.

### Corneal perforation with and without infection

The main cause of evisceration was corneal perforation (74.38%), with infectious and noninfectious keratitis. It was also the main cause of enucleation. Among the cases of corneal perforation without signs of infection, 60.46% were associated with glaucoma, and 20.93% were associated with painful blind eye that did not respond to clinical treatment with topical cycloplegics and corticosteroids. The patients who had infectious keratitis with corneal perforation and a visual acuity of NLP underwent evisceration (76.54%) or enucleation (23.46%). Twenty-five patients initially underwent clinical treatment to avoid surgery. These patients were treated with topical antibiotics, and 30.86% were also treated with cyanoacrylate glue and a bandage contact lens after corneal perforation. However, no clinical approach was successful.

Corneal scraping was performed before the surgical procedure in 27 (33.33%) of the 81 patients with infectious keratitis. On the basis of the culture results, 24 were positive for bacteria (60% gram positive and 40% gram negative); no microbial growth was observed in the remaining 3 patients. For one patient who underwent corneal scraping, the culture results were positive for two different bacteria. No positive results were obtained for fungi or *Acanthamoeba*. Coagulase-negative *Staphylococci* was the main gram-positive bacterial agent isolated (46.66%), and *Serratia marcescens* was the main gram-negative bacterial agent isolated (30%). Of the patients with perforated microbial keratitis, 38 (46.91%) had a history of glaucoma, and 21 (25.92%) had a history of painful blind eye.

### Endophthalmitis

The third leading cause of evisceration or enucleation was endophthalmitis, which was documented in 28 (15.38%) of the 182 patients. Half of these patients were treated with evisceration, and the other half were treated with enucleation. The main etiology of endophthalmitis was corneal ulcer (32.14%).

Seven patients (25%) with endophthalmitis were first treated with intravitreal injection of antibiotics, and one patient was treated with early pars plana vitrectomy. However, all these patients ultimately had a visual acuity of NLP.

The other causes of endophthalmitis were cataract surgery with complications (14.28%), endogenous source (7.14%), previous ocular trauma (7.14%), blebitis (3.57%), glaucoma tube shunt infection (3.57%), and postoperative corneal laceration repair (3.57%). The medical records of eight patients (28.57%) did not have enough information about etiology.

### Ocular trauma

Ocular trauma was the indication for enucleation or evisceration in 26 patients. Most patients (57.7%) were treated with evisceration rather than enucleation (42.3%). The ocular trauma was due to closed or open globe injury in 42.3% and 34.62% of the patients, respectively. Information on the mechanism of trauma was not available for the remaining 23% of the patients. In four and five patients, the open globe injury was caused by firearms (44.44%) and sharp weapons (55.56%), respectively.

Nineteen patients (73.07%) with ocular trauma presented a visual acuity of NLP at the initial examination. Only one patient had a visual acuity of LP, but the ocular globe could not be repaired because of the severity of the trauma. No information about the initial visual acuity was available in six patients (23.07%). Most patients who had an ocular trauma treated with evisceration or enucleation were male (84.61%), and only four (15.39%) were female.

### Neoplasia, burn injuries, and phthisis bulbi

Neoplasia was suspected in only one patient, who underwent enucleation. The patient had a visual acuity of no light perception in the affected eye, with signs of athalamia and hyphema. The findings from the ocular ultrasonography performed at another ophthalmologic center were consistent with choroidal melanoma, and anatomopathological analysis confirmed the diagnosis. After discharge, the patient was referred to the oncology section.

The burn injuries were severe and caused by chemical products. One of the patients had corneal perforation and visual acuity of the NLP. Treatment with cyanoacrylate glue and a bandage contact lens was attempted without success. The other patient had anterior staphyloma and could not provide information on visual acuity because of a cognitive disorder. Only one eye was eviscerated because of *phthisis bulbi*; the patient complained of painful blind eye and had a history of previous untreated retinal detachment.

### Complications

Twenty-five patients (20.66%) who underwent evisceration had complications or complaints after the surgical procedure. Nine patients (36%) had conjunctival dehiscence and had to undergo another surgical procedure. One patient had orbital cellulitis and was treated with intravenous antibiotic therapy and globe enucleation. Three patients had local infection treated with oral antibiotics. Two patients had sympathetic ophthalmia, and the remaining patients complained of pain or ocular secretion.

Both patients with sympathetic ophthalmia after evisceration had a history of previous trauma, and the complication occurred 4 months after evisceration in one patient and 5 months after evisceration in the other. One patient was 72 years old, and the other was 79 years old. Both were negative for syphilis and had normal complete blood count and chest radiographs. These examinations were requested to investigate differential diagnoses such as syphilis, lymphoma, tuberculosis, and sarcoidosis. The 72-year-old patient was lost to follow-up. The 79-year-old patient, who had undergone trabeculectomy in the sympathizing eye due to primary open-angle glaucoma, presented an increase in intraocular pressure after evisceration, which required a new trabeculectomy. Ocular inflammation control was achieved with systemic and topical corticosteroids, with a final visual acuity of 20/30.

Among the patients who underwent enucleation, one patient (1.64%) had conjunctival dehiscence, one complained of pain, and one complained of ocular secretion.

## DISCUSSION

Similar to previous studies^(3-5)^, our retrospective analysis revealed more eviscerations than enucleations. The main reasons for the greater proportion of eviscerations are the simpler nature of the technique and the inclusion criterion of only emergency procedures. Many surgeries at Hospital São Paulo are performed by medical residents in training, so evisceration is the preferred procedure if neoplasia is not suspected. None of the patients who underwent orbital computed tomography showed signs of intraocular neoplasia. Only one patient examined with ocular ultrasonography had signs consistent with choroidal melanoma. Another contributing factor to the smaller proportion of enucleations during the study period was the inclusion of patients who were not followed up by the ocular oncology division.

Although concerns about the possibility of sympathetic ophthalmia after evisceration have favored enucleation in the past, some authors have described low incidence rates of this complication and the safety of evisceration^(2,7,8)^. In our study, two patients (1.64%) had sympathetic ophthalmia after evisceration. Both patients were men aged >60 years who had a history of ocular trauma. Sympathetic ophthalmia is a rare complication that occurs mainly after cases of penetrating eye trauma. Intraocular surgery has become an important contributing factor to sympathetic ophthalmia due to the increase in the number of such procedures. Other reported contributing factors are non-penetrating trauma, cyclodestructive laser procedures, and radiotherapy^(8)^. Epidemiological data indicate that sex, age, or race had no dominant influence, but some studies have reported higher incidence rates in men, children, and individuals aged >60 years^(8)^.

The main indication for evisceration and enucleation surgery at our hospital was corneal perforation without visual prognosis. Glaucoma and painful blind eye were ocular conditions associated with corneal perforation with and without infection. Previous studies showed an association between glaucoma and severe microbial keratitis due to epithelial abnormalities or the use of glaucoma medications^(9,10)^.

The main microbial agents found in corneal scrapings in our study were *Streptococcus pneumoniae* and coagulase-negative *Staphylococci*. Green et al. showed that isolation of *Streptococcus pneumoniae* was associated with worse prognosis in infectious keratitis^(11)^. Delayed treatment was also described as a factor of worse prognosis by Cruz et al. and Titiyal et al.^(12,13)^ In the present study, the mean symptom duration before seeking ophthalmologic care was 18.32 days, indicating that treatment could have started earlier.

For patients with endophthalmitis, evisceration or enucleation was indicated owing to severity and the absence of visual prognosis. The main cause of endophthalmitis was corneal ulcer, which was previously reported to be a risk factor of evisceration or enucleation by Lu et al. and Tsai et al.^(14,15)^. As in other developing countries, our study shows that infectious causes are an important indication for destructive eye surgeries in Brazil^(16-19)^.

Ocular trauma is a preventable cause of eye injury that, along with infectious ocular conditions, represents one of the main causes of evisceration or enucleation in developing countries^(18,20-22)^. Consistent with prior reports, we found a higher frequency of ocular trauma among males than among females who underwent evisceration or enucleation^(18,20,21,23)^. Evisceration and enucleation were performed in 15 and 11 cases after ocular trauma, respectively. Previous studies indicated that the preference for enucleation to prevent sympathetic ophthalmia should be reconsidered because of the low risk of sympathetic ophthalmia and the benefits of evisceration in terms of motility and cosmetics^(24)^. In the present study, the cases of sympathetic ophthalmia after evisceration reported contradict these conclusions.

Overall, the number of serious cases of eye diseases at our academic hospital remained relatively constant during the study period. More eviscerations were performed more than enucleations, and male patients and individuals aged >60 years were the most common among our patients. The main indications for evisceration or enucleation procedures were corneal perforation with and without infection, endophthalmitis, and ocular trauma. Only one eye was enucleated because of intraocular neoplasia, which was diagnosed as choroidal melanoma after anatomopathological analysis. Two cases of sympathetic ophthalmia occurred after evisceration, which is a rare complication described in the literature. The large mean distance between a patient’s residence and the care provider shows the importance of investing in developing a structured health network. Public policies for specific awareness of eye trauma could be implemented to prevent trauma cases, and a larger network of ophthalmic coverage should be proposed to reduce cases of terminal illnesses such as perforated corneal ulcers with poor prognosis.
